# Choledochal cyst mimicker—When small bowel length matters

**DOI:** 10.1016/j.ijscr.2020.02.061

**Published:** 2020-02-29

**Authors:** Ammiel Arra, Nigel Bascombe, Barrie Landreth-Smith, Maria Bartholomew, Dilip Dan

**Affiliations:** a Department of Surgery, San Fernando General Hospital, San Fernando, Trinidad and Tobago; b Affiliated St. James Medical Complex, North West Regional Health Authority, St. James, Trinidad and Tobago; c FRCS RCPS Glasg., Affiliated Eric Williams Medical Sciences Complex, Mt. Hope, Trinidad and Tobago; d Affiliated Eric Williams Medical Sciences Complex, Mt. Hope, Trinidad and Tobago; e Department of Surgery, University of the West Indies, San Fernando General Hospital, San Fernando, Trinidad and Tobago; f Westshore Medical Limited, Cocorite, Trinidad and Tobago

**Keywords:** Case report, Choledochal cyst, Bile duct stones, Short bowel syndrome, Hepaticoduodenostomy

## Abstract

**Objective:**

Due to the risk of malignancy, the established management of choledochal cysts mandates bile duct excision and biliary reconstruction. While the reconstructive procedure of choice for most surgeons has traditionally been hepatico-jejunostomy, this may not be feasible in selected cases due to immobility or inadequacy of the jejunum. The following case will outline the management of a 32-year-old woman with short bowel syndrome, who was diagnosed with choledocholithiasis and a type 1 choledochal cyst.

**Method and materials:**

As a child, our patient suffered midgut volvulus secondary to malrotation which resulted in extensive bowel resection and developed short bowel syndrome. She presented with recurrent bouts of cholangitis. Imaging of her biliary tree confirmed common duct stones extending into the branched hepatic ducts, as well as a fusiform dilatation of the common bile duct, that appeared consistent with a type 1 choledochal cyst. Laparoscopic excision of the cyst with reconstruction using a hepatico-duodenostomy was planned.

**Results:**

The patient underwent successful laparoscopic cholecystectomy, CBD clearance with excision of the bile duct and reconstruction with hepatico-duodenostomy. Recovery was uneventful and she is asymptomatic on subsequent follow-up. Histology is consistent with a markedly dilated bile duct rather than a choledochal cyst.

**Conclusions:**

This case illustrates the dilemma of diagnosis and treatment of a dilated bile duct mimicking a choledochal cyst in the setting of short bowel syndrome and the feasibility of a laparoscopic approach in such cases. Also, it demonstrates that hepatico-duodenostomy may be a safe alternative in cases with limited material for conduit.

## Highlights

• The diagnosis and treatment of choledochal cysts (and conditions mimicking choledochal cysts) may prove difficult in patients with short bowel syndrome.
• Laparoscopic bile duct excision and reconstruction is a feasible and safe approach to bile duct excision in experienced hands.
• Hepatico-duodenostomy should be considered a safe alternative for biliary reconstruction in individuals with limited material for conduit.

## 1 Introduction

Laparoscopic techniques have been increasingly applied in recent years, although their use in complex hepatobiliary procedures has been constrained [1]. Among these include the resection of choledochal cysts, which are congenital dilatations of the biliary tree that result in recurrent infection, stone formation, and increased risk of malignancy [2]. Although various treatment modalities have been attempted, the current standard of management involves surgical excision followed by biliary reconstruction [3].

A variety of surgical reconstructive procedures have been described. The two most commonly used are Roux-en-Y hepatico-jejunostomy and hepatico-duodenostomy [4]. Although the former has been the approach traditionally favoured by surgeons, a dilemma may arise in patients who lack appropriate intestinal length [5]. Our case describes the management of a 32-year-old female patient with short bowel syndrome, who developed choledocholithiasis and dilatation of the common bile duct that appeared consistent with a choledochal cyst. This case was managed in a private care setting and reported in accordance with the SCARE Criteria [6].

## 2 Materials and methods

**Fig. 1:**
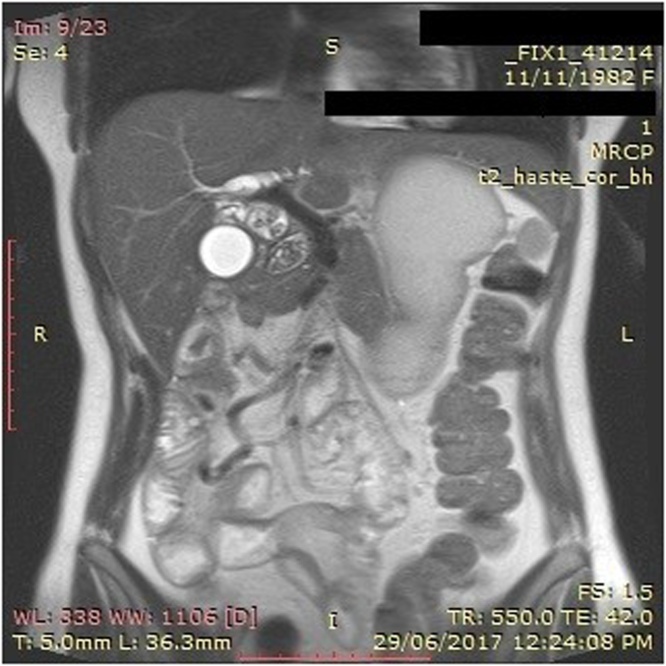
MRCP showing fusiform dilatation of the bile duct and multiple bile duct stones.

A 32-year-old woman presented to the hospital with a history of right upper quadrant pain and jaundice. She was diagnosed with gallstones a couple years before this presentation and was counselled on having cholecystectomy prior to pregnancy for which she was desirious. Interestingly, she had undergone extensive resection of gangrenous bowel as a child, secondary to intestinal malrotation and midgut volvulus. She remained with 100 cm of small intestine that was anastomosed to the transverse colon, and subsequently developed short bowel syndrome. Despite compensating well, she eventually developed cholelithiasis and extensive choledocholithiasis, resulting in recurrent bouts of acute cholangitis. On this admission, an ultrasound of the abdomen showed multiple stones in the gallbladder, as well as a dilated common bile duct measuring 1.8 cm in diameter. Imaging of her biliary tree identified common duct stones extending into the branched hepatic ducts, as well as a fusiform dilatation of the common bile duct, that appeared consistent with a type 1 choledochal cyst (Fig. 1).

Given these findings, a surgical approach was determined to be the best option to allow for excision of the cyst and biliary reconstruction. Although the preferred method of reconstruction was a Roux-en Y hepatico-jejunostomy, this was precluded by this patient's limited intestinal length. Therefore, laparoscopic cholecystectomy with clearance of the biliary tree, choledochal cyst excision and hepatico-doudenostomy was planned.

**Fig. 2:**
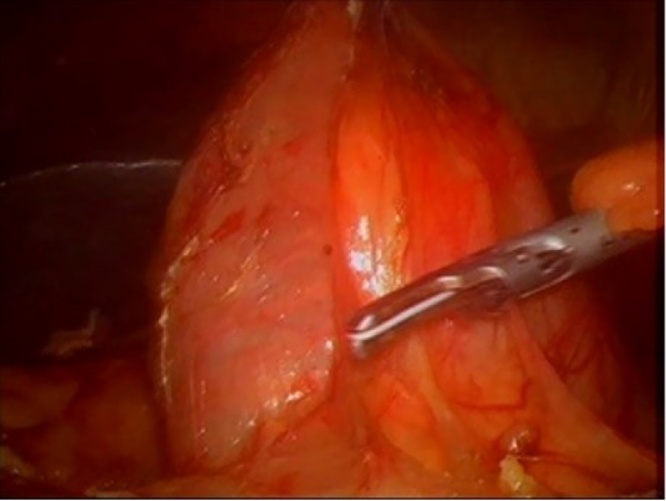
Kocherized Duodenum.

**Fig. 3:**
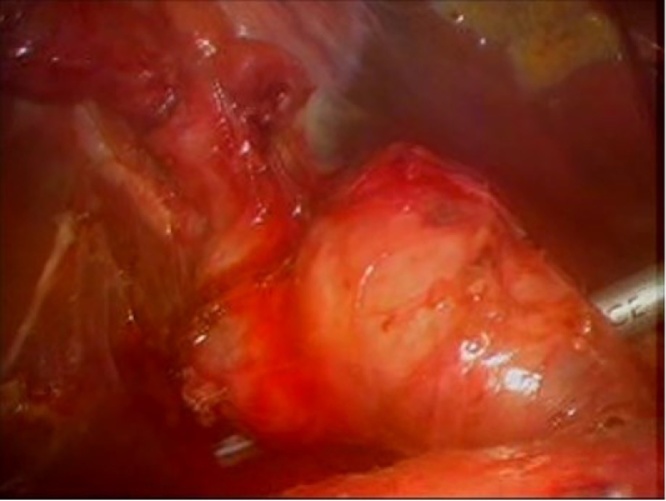
Dilated Common Bile Duct.

This was done with the patient in Lloyd-Davies position and the use of visual port entry at Palmer’s point. As expected, on achieving peritoneal access, significant adhesions were encountered and divided before identifying the gallbladder and proceeding with laparoscopic cholecystectomy. The critical view of safety (Strasberg) was attained, and the gallbladder dissected off the liver with the cystic structures intact. Cephalad retraction of the gallbladder faciliatated exposure of the hilar structures proximally and an extensive Kocher's manoeuvre was performed to mobilise the duodenum (Fig. 2). The mobility of the duodenum allowed for easy reach to the hilum. This was followed by circumferential dissection of the dilated common bile duct (Fig. 3).

**Fig. 4:**
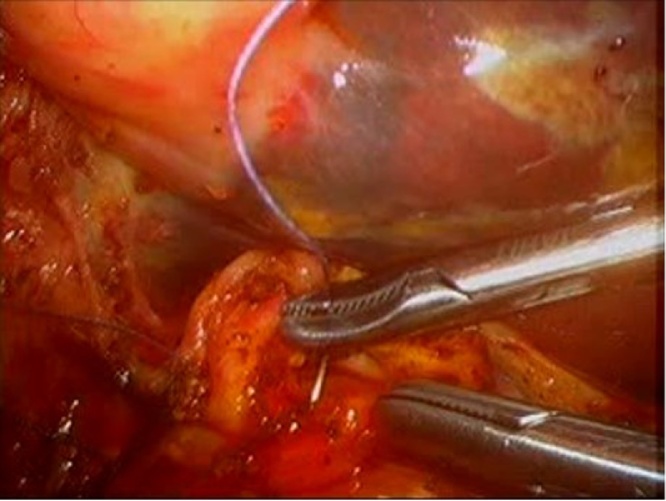
Intracorporeal Suturing of Hepaticoduodenostomy.

This dissection was extended to the intrapancreatic segment distally, at which point a transverse choledochotomy was done to extract the common bile duct stones. Clearance of the proximal and distal ducts was achieved by repeated flushing with saline and multiple passages of a fogarty catheter until clear. The proximal duct was divided at the hilar region, just below the confluence of the right and left hepatic ducts. The distal duct was transected with a linear stapler device. A tension-free hepaticoduodenostomy was performed using intra-corporeal suturing technique using interupted 3/0 absorbable Vicryl suture (Fig. 4).

A short video outlining the key aspects of the procedure can be accessed using the following link: https://1drv.ms/v/s!AkSawWgNOFMogb8d0YCJqqF2jeHXUQ.

The procedure lasted just under four hours, and the patient was discharged on day 3. She had an uneventful recovery and remains asymptomatic on subsequent follow-up. Despite the preoperative radiological findings, histological examination was consistent with a markedly dilated bile duct rather than a choledochal cyst. Written consent was obtained from the patient allowing for publication of this case and use of operative footage. IRB approval was not required in this case.

## 3 Discussion

The earliest attempt of biliary construction using laparoscopic techniques was a laparoscopic cholecysto-jejunostomy in 1992, although since then more advanced procedures have been attempted [7,8]. Laparoscopic choledocho-duodenostomy (CD) is the simplest of these, and several investigators including Rhodes and Tinoco et al. have reported success with this technique [9,10]. The management of choledochal cysts involves total resection of the bile duct due to their risk for malignant potential. Therefore, biliary reconstruction requires anastomosis to the hepatic duct, which is shorter and less accessible [3]. Tanaka et al. reported 5 cases of laparoscopic choledochal cyst resection with hand-assistance, while larger series from Patil and Santore et al. have performed up to 50 cases without hand assistance [4,11].

The most common methods of reconstruction for these cases are the Roux-en-Y hepatico-jejunostomy and the hepatico-duodenostomy. Although most surgeons favour the former due to a perceived lower complication rate, it may not always be possible as illustrated in our case. Furthermore, a review of the literature reveals that there is significant debate as to whether the concerns related to hepatico-duodenostomy are in fact warranted, with some authors suggesting that this should be the preferred method of reconstruction [[12], [13], [14], [15]].

A report by Todani et al. found no significant difference in early complications between the two procedures, although hilar cholangiocarcinoma was observed 19 years after hepatico-duodenostomy in one case [15,16]. Another study by Shimotakahara et al. compared 28 patients who underwent hepatico-jejunostomy with 12 patients who had hepatico-duodenostomy after choledochal cyst resection. Bile gastritis was observed in 4 of 12 hepatico-duodenostomy patients but was not apparent in hepatico-jejunostomy patients. Two hepatico-jejunostomy patients, however, developed postoperative adhesional bowel obstruction with no such instances in the hepatico-duodenostomy group [17]. Similar results were outlined by Takada et al. in a smaller series that described endoscopic evidence of bile gastritis even though patients were asymptomatic [18].

A large single centre study published in the Indian Journal of Surgery in 2012 reviewed the use of hepatico-duodenostomy for biliary reconstruction following choledochal cyst excision in 54 patients over a 25-year period. Most patients had an uneventful postoperative recovery, however, three episodes of biliary leak were noted which settled with conservative management. An anastomotic stricture was observed in one patient 18 years after the initial procedure. In this series, hepatico-duodenostomy was appraised as a relatively safe procedure with low complication rates [11]. A meta-analysis including six retrospective studies and 679 patients compared the two methods and found that the hepatico-duodenostomy group had shorter hospital stay but showed a higher incidence of postoperative reflux. When comparing operative time and need for re-intervention, no difference was identified between hepatico-duodenostomy and hepatico-jejunostomy groups. Also, rates of bile leak, cholangitis, stricture and adhesive intestinal obstruction were similar [19].

Our case posed several unique challenges and presented both diagnostic and therapeutic dilemmas. In a patient who has presented with complicated gallstones disease and recurrent episodes of cholangitis, the ideal management would usually comprise endoscopic clearance of the bile duct followed by laparoscopic cholecystectomy. The presence of multiple stones extending proximally into the hepatic ducts of this patient precluded endoscopic clearance in favour of a surgical drainage procedure such as a choledocho-duodenostomy. The fusiform dilatation of the common bile duct, however, further complicated matters, mandating excision of the apparent choledochal cyst and biliary-enteric reconstruction. Although hepatico-duodenostomy was successfully used in this case, some may argue that its possible long-term consequences make it a less attractive option for younger patients with longer life expectancy.

However, in such cases where limited intestinal length may prevent other methods of reconstruction, hepatico-duodenostomy is a viable option. When compared to hepatico-jejunostomy, it requires a single anastomosis and is easier to perform. In addition to providing a better physiological result, it also preserves endoscopic access to the biliary tree which we feel is a great advantage not only if a leak or stricture occurs but also for surveillance and biopsy. A literature search for similar cases was performed, and 1 case was discovered, performed by Tan et al. using similar techniques, at the University Kebangsaan Malaysia Medical Centre, which was published in 2011 [5].

## 4 Conclusion

This case illustrates the dilemma of diagnosis and treatment of choledochal cysts (and conditions mimicking choledochal cysts) in patients with short bowel syndrome. Similar to other studies, laparoscopic bile duct excision and reconstruction was found to be feasible, and no complications were observed in the early postoperative period. Despite the theoretical long-term risk of anastomotic stricture, the preserved access afforded by hepatico-duodenostomy facilitates endoscopic intervention. Therefore, hepatico-duodenostomy should be considered a safe alternative in selected cases with limited material for conduit.

## Funding

This submission is a case report and therefore no funding was required.

## Ethical approval

Exemption of ethical approval was provided by the institution.

## Consent

Written consent from the patient was obtained for publication of this case report.

## Author contribution

Dr. Ammiel Arra – Primary author and editor of video.

Dr. Dilip Dan/Dr. Nigel Bascombe/Dr. Barrie Landreth-Smith – Surgeons involved in patient’s management. Provided review of article prior to submission.

Dr. Maria Bartholomew – Gastroenterologist involved in patient’s care. Provided review of article prior to submission.

## Registration of research studies

Article submitted was a case report and therefore not applicable.

## Guarantor

All authors were involved in the preparation of this case report.

Primary Surgeon – Prof. Dilip Dan.

## Provenance and peer review

Not commissioned, externally peer-reviewed.

## Declaration of Competing Interest

None of the authors have any conflicts of interest to declare.
